# Relative Late Gestational Muscle and Adipose Thickness Reflect the Amount of Mobilization of These Tissues in Periparturient Dairy Cattle

**DOI:** 10.3390/ani11082157

**Published:** 2021-07-21

**Authors:** Conor McCabe, Aridany Suarez-Trujillo, Theresa Casey, Jacquelyn Boerman

**Affiliations:** Department of Animal Sciences, Purdue University, West Lafayette, IN 47907, USA; mccabec@purdue.edu (C.M.); asuarezt@purdue.edu (A.S.-T.); tmanders@purdue.edu (T.C.)

**Keywords:** tissue mobilization, nutrient partitioning, late gestation

## Abstract

**Simple Summary:**

One of the main characteristics of the periparturient period is the mobilization of adipose and muscle reserves to support the metabolic demands of fetal growth and lactation. A comparison of cattle with low vs. high *longissimus dorsi* thickness in late gestation demonstrated that high-muscle cows gained additional backfat, whereas cattle with less muscle thickness gained additional muscle, from approximately one month prior to calving through calving. High-muscle cows subsequently mobilized more muscle and fat than low-muscle cows and yielded less milk through 60 days in milk. Thereby, the relative amount of muscle mass in late gestation may be related to metabolic strategies that support the fetus and milk production during the periparturient period.

**Abstract:**

Due to insufficient dry matter intake and heightened nutrient requirements in early lactation, periparturient dairy cows mobilize adipose and muscle tissues to bridge energy and amino acid gaps, respectively. Our objective was to evaluate the relationship between the relative muscle thickness of late pregnant cows and their early lactation performance. At 35 d before expected calving (BEC), *longissimus dorsi* muscle thickness (LDT) was measured in forty-one multiparous Holstein cows via ultrasound. Tissue mobilization was evaluated via ultrasound images of LDT and backfat thickness (BFT) at 21 and 7 d BEC as well as at 0, 10, 30, and 60 DIM. Plasma concentrations of 3-methylhistidine (3-MH), creatinine (CRE), non-esterified fatty acids (NEFA), and β-hydroxybutyrate (BHB) were evaluated weekly. Milk yield and milk component data were collected through 60 DIM. Cattle were assigned post hoc to high-muscle (HM; *n* = 20; LDT > 4.49 cm) or low-muscle (LM; *n* = 21; ≤4.37 cm) groups, with mean LDT at 35 d BEC greater in HM (5.05 ± 0.49) than in LM (3.52 ± 0.65) animals. Between 35 and 21 d BEC, LM cows gained LDT, whereas HM cows gained BFT. HM cows mobilized more muscle from 21 d BEC to 30 DIM, as reflected by a greater loss of LDT, greater 3-MH concentrations (532 vs. 438 ± 30 ng/mL), and a greater 3-MH:CRE ratio (0.164 vs. 0.131 ± 0.008) in the first three weeks postpartum. The LDT and BFT at 21 d BEC were related to the amount of respective tissue mobilized through 30 DIM (R^2^ = 0.37 and 0.88, respectively). Although calves born to HM cattle were larger (45.2 vs. 41.8 ± 0.7 kg), HM cows produced less milk (38.8 vs. 41.6 ± 0.8 kg/d) with a tendency towards higher fat content (4.33 vs. 4.05 ± 0.12%), likely related to the mobilization of more backfat from 0 to 60 DIM (1.78 vs. 0.68 ± 0.34 mm), compared to LM cattle. These findings suggest that a cow’s metabolic status, as measured by LDT and BFT prepartum, may influence the metabolic strategy the animal uses to meet energy and amino acid requirements in late gestation and early lactation.

## 1. Introduction

In dairy cattle, physiological adaptations from gestation to lactation are marked by coordinated changes in metabolism, partitioning nutrients in order to support the developing fetus and the subsequent demands of milk synthesis [1]. The coordinated changes in metabolism ensure that the cow can accommodate the physiological demands of late gestation and the onset of lactation. The metabolic adaptations to be able to support the additional energetic and nutrient demands include increased tissue mobilization [2], increased liver gluconeogenesis [3], and reduced peripheral insulin sensitivity [4].

In early lactation, energy and amino acid (AA) gaps result from elevated nutrient requirements coupled with the cow’s inability to consume adequate dry matter. To overcome this deficit, cattle mobilize adipose and muscle tissue reserves to meet glucose and AA requirements in fetal growth, mammogenesis, and lactation. High rates of adipose and muscle mobilization in cattle were reported to be initiated as long as ten days prior to parturition [5,6]. Tissue mobilization is subsequently heightened at calving with the spike of glucocorticoids, promoting the mobilization of substrates from tissues [7] and continues throughout the early post-parturition period via a reduction in circulating insulin concentrations [8]. Increased mobilization is associated with a further reduction in insulin sensitivity [9]. In Holstein multiparous cows weighing in excess of 725 kg prepartum, a mean of 54 kg of body fat and 21 kg of protein were estimated to be mobilized from two weeks prior to parturition until five weeks in lactation [2]. However, a significant variation between animals was observed in the amount of tissue mobilized [2,10]. Excessive tissue mobilization contributes to metabolic disease development during the periparturient period, which impacts the welfare of cattle and economic returns to the producer [11]. An improved understanding of the metabolic changes that occur over the periparturient period and the environmental stressors that affect these changes can aid in the development of management strategies that mitigate excessive tissue mobilization [12].

Several experiments demonstrate that cows mobilize adipose tissue in early lactation relative to the amount of adipose tissue they have in late gestation [13,14]. However, there are a limited number of studies that have examined the relationship between the amount of relative muscle in late gestation dairy cows and the amount of muscle and adipose tissue mobilized through the transition period. We assigned cattle to either a low or high muscle group depending on their *longissimus dorsi* thickness (LDT) in late gestation and measured LDT and back fat thickness (BFT) biweekly, from 5 weeks before expected calving (BEC) to 2 months postpartum. We hypothesized that cows with greater LDT would mobilize more muscle tissue approximately five weeks prior to calving. A greater change in muscle thickness was surmised to be associated with greater milk production and with a greater milk protein yield through 60 days in milk (DIM). The liberation of AA from protein reserves would increase the supply of metabolizable protein, which has been associated with both increased milk protein and milk production in early lactation. Therefore, our study objectives were to determine if dairy cows with high versus low LDT in late gestation promoted differences in muscle and adipose tissue mobilization, milk production, and dry matter intake through the first two months of lactation.

## 2. Materials and Methods

### 2.1. Animal Management and Experimental Design

Data for this study were collected over two experiments performed at Purdue University Animal Science Research and Education Center (ASREC) dairy unit. All procedures described were reviewed and approved by IACUC protocol #1701001523 prior to the start of experiments. The first experiment took place from January to July 2018. Data were collected from 25 multiparous cows with 2.92 ± 1.19 (mean ± SD) lactations, a milk yield of 12,569 ± 1299 kg in the previous lactation, and a body weight of 766 ± 102 kg at experiment enrollment. The second experiment took place from February to June 2019. Data were collected from 16 multiparous cows with 2.88 ± 0.91 lactations, a previous lactation milk yield of 12,277 ± 3460 kg, and a body weight of 752 ± 66 kg at five weeks BEC. In both experiments, cows were blocked by lactation number, previous lactation yield, and number of disease events (hyperketonemia, mastitis, hypocalcemia, metritis, retained placenta, and displaced abomasum; data previously reported [15,16]) during the previous lactation, into one of two treatments—control (CON) or phase shift (PS) at 35 d BEC. From 35 d BEC through calving, CON animals received a consistent timing of 16 h of light and 8 h of dark every day, whereas PS cows received the same amount of light in a 6 h forward shift of the photophase every 3 d. Then from calving through 60 DIM, all cattle received the CON light–dark cycle. Further details on CON and PS treatments, experimental procedures, and power analysis for this study design are available in our previous manuscripts [15,16]. No differences in LDT or BFT changes were observed between CON and PS cattle.

At the time of experiment enrollment, 35 d BEC (32.0 ± 3.5 d before calving), images of LDT and BFT were captured of all animals using an Aloka SSD-500 ultrasound (Wallingford, CT, USA). The *longissimus dorsi* muscle and backfat location were selected for ultrasound scanning due to evidence that the respective tissue thickness is highly correlated with total body protein and adipose tissue [17,18,19]. Ultrasound measurements of BFT and LDT were also taken at 21 and 7 d BEC, and at 0, 10, 30, and 60 DIM. Ultrasound images of the right side of each animal at the 12th intercostal space were captured by trained individuals in each experiment using methods adopted from [20]. Three images were collected at each time point of LDT and BFT measurements using methods described by [21] for the collection of images of a standing animal without tissue compression. LDT was subsequently measured in cm, while BFT was measured in mm using ImageJ software (NIH, Bethesda, Rockvill, MD, USA). The resulting three measurements of each time point were averaged. Measurements across the three replicates with an intra CV greater than 15% were removed as outliers.

When tissue measurements were compared between PS and CON at each time point, there was no effect of treatment. The cows were then assigned post hoc into low and high LDT groups based on measurements at experiment enrollment (35 d BEC) to evaluate the relationship of prepartum LDT and BFT with calf birth weight, feed intake, early lactation performance, and metabolic health parameters. Cows that had a LDT > 4.49 cm at experiment enrollment (35 d BEC) were assigned to the high-muscle (HM) group (*n* = 20; 5.05 ± 0.49 cm, range 4.49–6.50 cm), whereas cattle with a LDT < 4.37 cm at enrollment were assigned to the low-muscle (LM) group (*n* = 21; 3.52 ± 0.65 cm, range 2.04–4.37 cm). Initial muscle depth was different between groups (*p* < 0.05). The HM and LM groups were similar in lactation number (3.05 ± 0.89 vs. 2.82 ± 1.09) and treatment (11 CON, 9 PS vs. 8 CON, 13 PS), but different in experiment number distribution (15 first experiments, 5 s experiments vs. 10 first experiments, 11 s experiments). There was no difference in previous lactation milk yield between HM and LM cows (12,589 vs. 12,713 ± 337 kg; *p* > 0.05).

### 2.2. Feed Intake

Cows were fed *ad libitum* TMR that was formulated to meet or exceed all nutrient requirements according to [22], from 35 d BEC through 60 DIM. Feed intake of individual cows was measured daily from 35 d BEC until approximately 15 DIM, when cows were moved to the free stall barn. Animals were fed *ad libitum* for 10% refusals from 35 d BEC to 15 DIM. In both experiments in the prepartum period, cows were fed at 1600 h each day. In the postpartum period in experiment 1, cows were fed at 0800 h and in experiment 2 at 1600 h each day. Feed ingredient samples were collected every two weeks and analyzed for nutrient composition. Samples were dried at 60 °C for 48 h and ground through a 1 mm mill (Retsch GmbH, Haan, Germany). Feed samples were measured for neutral detergent fiber and acid detergent fiber [23] (Ankom, Macedon, NY, USA). Percent ash content was determined by contents remaining after a 24 h oven cycle at 600 °C. Crude protein was determined based on analysis via pure nitrogen concentration (LECO, St. Joseph, MI, USA).

In both trials, all cows were fed a similar prefresh and lactating diet (Table 1). The forage in the prefresh diet was composed of corn silage, rye grass hay or wheat straw, and a mix of mostly legume grass silage. On the other hand, the lactation diet consisted of corn silage, a mix of mostly legume silage, high moisture corn, soybean meal, and a vitamin and mineral premix supplement. The prefresh diet consisted of 51–53% DM, 15.9–16.9% CP, 35.5–37.5% NDF, 20–24% ADF, 5–8% ash, and 25% starch. The lactating diet consisted of 51–53% DM, 13.8–14.2% FCP including rumen-protected lysine and methionine, 22–27% NDF, 16–18% ADF, 5–8% ash, and 27–29% starch.

### 2.3. Body Weights and Body Condition Scoring

Body measurements of each animal were taken at −35, 0, 30, and 60 d relative to calving. Cattle were weighed directly following the morning milking and within one hour after calving on the day of parturition. At calving, calf sex was recorded, and calves were weighed prior to being fed colostrum. Body condition scoring (BCS) was also performed on all cows by three trained researchers and the scores were averaged at −35, 0, 30 and 60 d relative to calving using the five-point scoring system with quarter point increments [24].

### 2.4. Milk Sampling and Analysis

Animals were milked twice daily at 0500 h and 1600 h. In both studies, 50 mL milk samples from each milking were collected at 7, 14, 21, 30, and 60 DIM. Individual milk samples were analyzed separately and averaged by day for protein, fat, milk urea nitrogen, and lactose using Fourier Transform Infrared Spectroscopy (MilkoScan 7 RM, Foss, Hillerrød, Denmark) at Dairy One (Ithaca, NY, USA). Respective daily component yields were calculated by multiplying the component percentage by the milk weight, subsequently summing the product of the morning and afternoon milk weights. Component percentages were determined by dividing the daily component yield by the daily milk yield.

### 2.5. Blood Sampling and Metabolite Analysis

In both experiments, blood samples from the coccygeal vessels were collected into 10 mL K_2_ EDTA tubes, between 0430 and 0530 h (Becton Dickinson, Franklin Lakes, NJ, USA). Blood was collected at 35, 28, 21, 14, and 7 d BEC and at 2, 7, 14, and 21 DIM. Within 60 min of blood collection, tubes were centrifuged at 4000× *g* for 15 min at 4 °C, and aliquots of plasma were frozen at −20 °C until further analysis. At the same time points, blood was collected from the coccygeal vessels using a syringe (Covidien; Dublin, Ireland) and a 16-gauge needle (Becton Dickinson, Franklin Lakes, NJ, USA) for a whole blood sample. This sample was then immediately analyzed for glucose and β-hydroxybutyrate (BHB) using the Centrivet meter (ACON Laboratories; San Diego, CA, USA), as described by [25] with BHB concentrations in whole blood samples.

Plasma samples were analyzed for creatinine (CRE) and 3-methylhistidine (3-MH) at 35 and 21 d BEC as well as 2, 7, 14, and 21 DIM. The metabolites were analyzed via liquid chromatography–tandem mass spectrometry (LC–MS/MS). In short, 500 μL of plasma were extracted using acetonitrile (1:4 *v/v*), vortexed and centrifuged at 4000× *g* for 10 min. The supernatant was collected, vacuum-dried, and stored at −80 °C until further analysis. Chromatography was performed using an Imtakt Intrada Amino Acid 3 µm 2 × 150 mm column (Chrom Tech Inc., Apple Valley, MN, USA), as described and modified by [26]. Plasma CRE concentrations were determined via an internal spiked marker of labeled CRE-d_3_ (Sigma Aldrich; St. Louis, MO, USA) and 3-MH was determined via a standard curve.

Plasma samples from 35 and 21 d BEC and from 2, 7, 14, and 21 DIM were analyzed for insulin. Additionally, samples collected at 35, 21, and 7 d BEC and at 2, 7, 14, and 21 DIM were analyzed for NEFA. Bovine insulin was measured using a bovine insulin ELISA kit following the manufacturer’s approach (ALPCO; Salem, NH, USA), and NEFA was measured using a commercial kit (WAKO; Mountain View, CA, USA). Intra-assay CVs were 3.44% and 5.06% for NEFA and insulin, respectively. Inter-assay CVs were 3.52% and 16.94% for NEFA and insulin, respectively.

### 2.6. Statistical Analysis

A post hoc power analysis was performed using data from these experiments where an LDT change of 0.99 cm, with a standard deviation of 0.96 cm, was observed from 35 d BEC through 60 DIM. Our sample size of 41 cows (*n* = 20 and *n* = 21 per group) was found to have a power of 0.90.

Data were analyzed using the MIXED Procedure of SAS 9.4 (Cary, NC, USA). Measurements for LDT at all time points were analyzed for normality using a Shapiro–Wilk test, and data were normally distributed. Our expectation was that metabolites and hormones would not be normally distributed because of our sampling times relative to parturition, especially for metabolites such as NEFA, which varies by day relative to parturition. Data were screened for influential outlying data points using a cutoff; Cook’s D values > 0.3 were removed (*n* ≤ 1) for each dataset.

The model used for analysis is as follows:

Y_ijkl_ = µ + G_i_ + D_j_ + G × D_ij_ + C(G)_k(i)_ + T + N + L + e_ijk_ (1)

where Y_ijkl_ is the dependent variable, µ is the overall mean, G is the fixed effect of group (i = HM or LM), D is the fixed effect of day of study (j = 35 d BEC to 60 DIM), G × D is the interaction between the fixed effect of group and the fixed effect of day of study, C(G)_k(i)_ is the random effect of cow nested within treatment, T is the covariate of treatment (CON or PS), N is the covariate of experiment number (1 or 2), L is the covariate of lactation number (2 or 3+), and e_ijk_ is the random error term. The covariates of the previous treatment, the experiment number, and the lactation number were initially included in the model to control for the effect of treatment on evaluated variables, the variation between experiments, and the variation between cows of different parity. All metabolites and hormone data were separated between the pre- and postpartum time points to determine the effect of tissue thickness during the prepartum on postpartum performance. For BCS, BW, LDT, and BFT, a one-way ANOVA test was performed. Calf birthweight was evaluated by including the covariate of calf sex along with the fixed effect of group, and the covariates of experiment number, treatment, and lactation number. When analyzing all data, if a covariate had a *p*-value > 0.20 from the initial analysis, it was removed from the model for that analysis to simplify the model given that the interpretation of results remained the same when covariates with *p*-values > 0.20 were removed. Data was considered significant at *p* ≤ 0.05, and trends were indicated if the *p*-value was ≤0.10 and >0.05.

## 3. Results

### 3.1. Daily Feed Intake

All cows were fed the diets *ad libitum* during the prepartum and postpartum periods. There were no differences in daily dry matter intake (DMI) between the LM and HM groups (Figure S1).

### 3.2. Tissue Mobilization as Assessed by Ultrasound Evaluation

**Figure 1:**
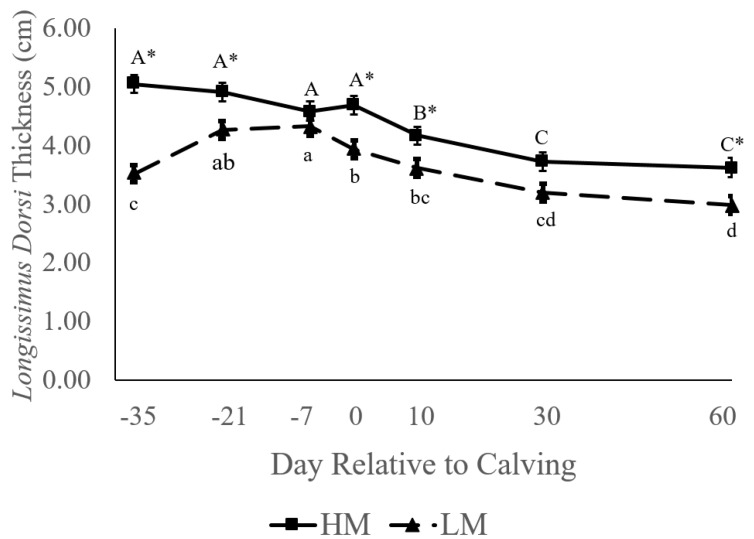
*Longissimus dorsi* thickness (LDT) between high-muscle (HM; *n* = 20) and low-muscle (LM; *n* = 21) cows at different time points, from 35 d before expected calving (BEC) to 60 d in milk (DIM). Cows were assigned to a group based on LDT at 35 d BEC. HM LDT was >4.49 cm and LM LDT was ≤4.37 cm at 35 d BEC. Differences in uppercase letters indicate a difference between time points within HM; lowercase letters indicate a difference between days within LM; and an asterisk indicates a difference between groups within a day at *p* < 0.05.

High-muscle cows had greater *longissimus dorsi* muscle thickness than LM cows at several time points from 35 d BEC through 60 DIM (Figure 1; *p* < 0.05). Between 35 d BEC and calving, there was no change in muscle thickness in HM cows, whereas the LM group exhibited a significant gain in muscle thickness between 35 d and 21 d BEC (Figure 1). The LM group subsequently initiated a loss of muscle thickness between 7 d BEC and parturition. Both the HM and LM groups lost muscle from calving to 60 DIM (Table 2). However, HM cows began losing LDT between 0 and 10 DIM, whereas LM cows first lost LDT between 7 d BEC and 0 DIM.

**Figure 2:**
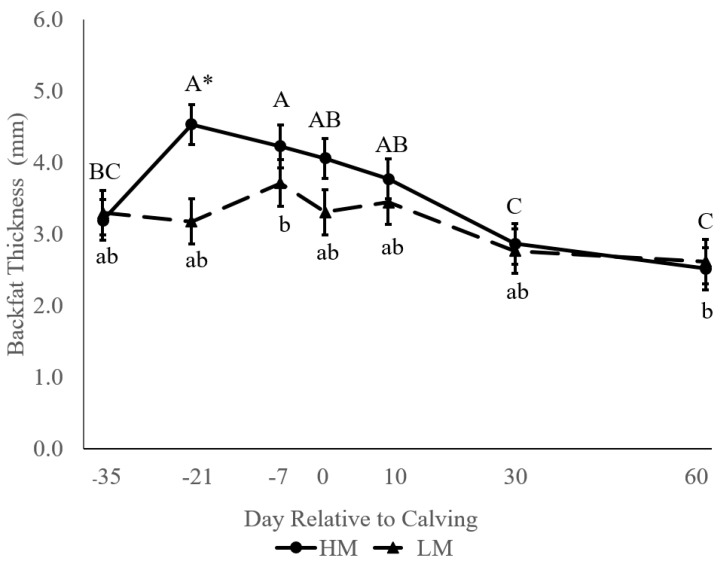
Backfat thickness (BFT) between high-muscle (HM; *n* = 20) and low-muscle (LM; *n* = 21) cows at different time points, from 35 d before expected calving (BEC) to 60 d in milk (DIM). Cows were assigned to a group based on *longissimus dorsi* thickness (LDT) at 35 d BEC. HM LDT was >4.49 cm and LM LDT was ≤4.37 cm at 35 d BEC. Differences in uppercase letters indicate a difference between time points within HM; lowercase letters indicate a difference between days within LM; and an asterisk indicates a difference between groups within a day at *p* < 0.05.

At 35 d BEC, there was no difference in BFT between groups (3.20 vs. 3.30 ± 0.33 mm; Figure 2). However, HM cows gained BFT between 35 d BEC and 21 d BEC, resulting in HM animals having greater BFT than those in the LM group at three weeks prepartum (Figure 2). Between 21 d BEC and 60 DIM, HM cows mobilized more BFT than LM cows, resulting in a tendency towards a group by timepoint interaction (*p* = 0.07). No difference was observed in BFT between groups from 7 d BEC onwards. The greatest change in BFT for all cows was observed between 0 and 30 DIM, relative to differences of 35 d BEC–0 DIM and 30–60 DIM.

### 3.3. Body Weight and Body Condition

At 35 d BEC and calving, HM cows weighed more than LM cows (Table 2). There was no difference in BW or change in BW between groups at 30 and 60 DIM. HM and LM cows lost significant BW from 35 d BEC through 60 DIM. High-muscle cows also had greater BCS at 35 d BEC and calving (*p* < 0.05), and both groups lost significant BCS from 35 d BEC through 60 DIM. The largest BCS losses were observed from 0 to 30 DIM as compared to 35 d BEC–0 DIM and 30–60 DIM, respectively (*p* < 0.05).

### 3.4. Blood Metabolites and Insulin

The prepartum and postpartum periods were analyzed separately due to differences in nutrient requirements between late gestation and early lactation [8]. High-muscle cows exhibited lower glucose concentrations during the five weeks BEC (76.8 vs. 79.8 ±0.9 mg/dL; *p* = 0.03) and a tendency towards greater CRE (3328 vs. 3086 ± 94 ng/mL; *p* = 0.06) and 3-MH (453 vs. 395 ± 29 ng/mL; *p* = 0.09) at five and three weeks prior to calving (Table 3). There were no differences in any of the other metabolites or insulin concentrations between groups during the prepartum time points measured (Table 3). In the postpartum period, there were no differences between the groups in insulin, BHB, glucose, or NEFA concentrations (*p* > 0.05; Table 4). High-muscle cows had higher 3-MH concentrations (533 vs. 438 ± 30 ng/mL; *p* = 0.02) and had a greater 3-MH:CRE ratio (0.164 vs. 0.131 ± 0.008; *p* < 0.01) as compared to LM cows in the first three weeks postpartum.

### 3.5. Cows Mobilized Tissue According to Tissue Thickness at Enrollment

**Figure 3:**
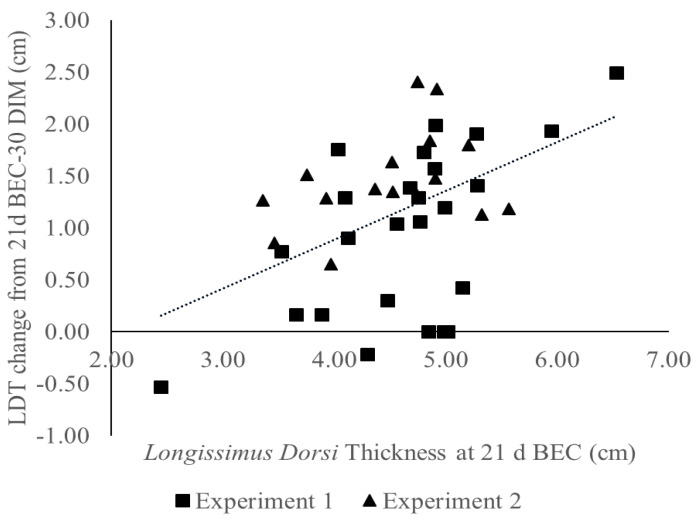
The relationship between *longissimus dorsi* thickness (LDT) at 21 d before expected calving (BEC) and the amount of LDT mobilized from 21 d BEC to 30 days in milk (DIM) from the first experiment (∎; *n* = 25) and the second experiment (▲; *n* = 16). The greater the LDT cows have at 21 d BEC, the more LDT they mobilize from three weeks prepartum through 30 DIM (R^2^ = 0.37). Negative LDT mobilized is equivalent to muscle accretion over the time points measured.

A linear regression analysis was used to determine if there was a relationship between muscle or adipose tissue thickness and a change in tissue thickness during the transition period. Muscle and adipose tissue accretion occurred between 35 d BEC and 21 d BEC, resulting in maximal thickness for both groups at 21 d BEC, whereas the lowest tissue thickness was realized at 30 DIM for both groups. Thus, the relationship between tissue thickness at 21 d BEC and the change in tissue thickness between 21 d BEC and 30 DIM was used in the regression analysis as they represented the peak and nadir of LDT and BFT. A positive relationship was found between LDT at 21 d BEC and the amount of muscle tissue mobilized through 30 DIM (R^2^ = 0.37; Figure 3).

**Figure 4:**
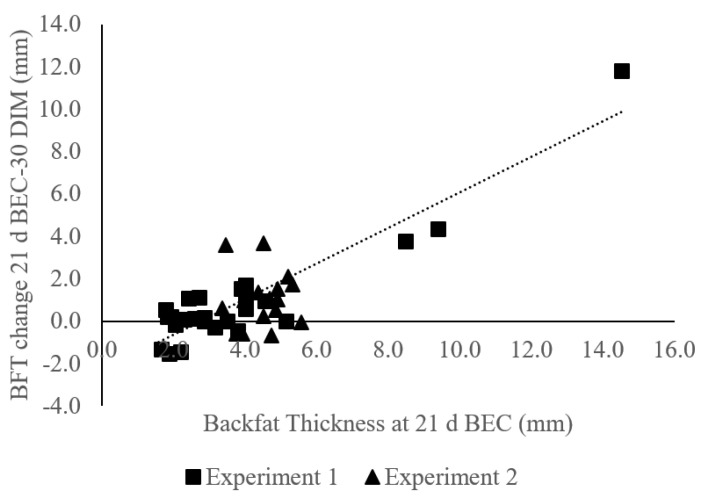
The relationship between backfat thickness (BFT) at 21 d before expected calving (BEC) and the change in BFT from 21 d BEC to 30 days in milk (DIM) from the first experiment (∎; *n* = 25) and second experiment (▲; *n* = 16). The greater amount of BFT cows have at 21 d BEC, the more BFT they mobilize from three weeks prepartum through 30 DIM (R^2^ = 0.87). Negative BFT mobilized is equivalent to adipose accretion over the time points measured.

While the percentage of muscle tissue mobilized varied between cows (Range: 50% loss to 22% gain), on average, cows mobilized 1.25 ± 0.68 cm or 26.5% of their LDT present from three weeks prepartum through 30 DIM. There was also a positive relationship between BFT at 21 d BEC and the amount of BFT mobilized from 21 d BEC through 30 DIM (R^2^ = 0.87; Figure 4), and a large range in BFT mobilization (81% loss to 81% BFT gain). On average, cows mobilized 1.05 ± 2.26 mm or 15.2% of their BFT at 21 d BEC through 30 DIM.

### 3.6. Production Outcomes

All cows gave birth to a single calf. High-muscle cows (11 F; 9 M) gave birth to heavier calves as compared to LM cows (8 F; 13 M) (45.2 vs. 41.8 ± 0.7 kg; *p* < 0.01; Table 5). Additionally, HM cows produced less milk per day than LM cows, from 1 to 60 DIM (38.8 vs. 41.6 ± 0.8 kg/d; *p* < 0.05). HM cows had a tendency to have greater milk fat concentrations across the five time points sampled (4.33 vs. 4.05 ± 0.12%; *p* = 0.09). However, there were no differences in milk protein or lactose concentrations for the time points that milk composition was measured (*p* > 0.05). Similarly, there were no differences between groups in lactose, protein, and fat yield; there was also no difference in milk urea nitrogen concentrations (*p* > 0.05).

## 4. Discussion

Across all cows, LDT decreased from three weeks BEC to a study nadir at 30 DIM. There was a linear relationship between LDT and BFT in late gestation and the amount of muscle and fat that cows mobilized, respectively, through 30 DIM. The profiles of muscle and fat accretion and mobilization were distinct between cattle assigned to high versus low muscle thickness groups. Between five and three weeks BEC, the high-muscle group had no change in muscle but accreted a significant amount of fat, whereas the low-muscle group accreted muscle but had no change in BFT. These distinct patterns resulted in the animals in the high-muscle group gaining greater muscle and fat thickness at three weeks BEC. Moreover, although the start of mobilization of the *longissimus dorsi* muscle was earlier in animals in the LM group, the HM group mobilized more muscle and more fat through 30 DIM. Consistent with greater loss in LDT, HM cows exhibited greater 3-MH concentrations and a greater 3-MH:CRE ratio in the postpartum period, thus supporting they mobilized more protein and mobilized more protein per unit of total body protein. Together, these findings indicate that animals with high versus low muscle tissue thickness used different sources of nutrients to meet the energetic and AA demands of the transition period.

### 4.1. Longissimus Dorsi Thickness (LDT) Impact on Muscle Mobilization

Our hypothesis that cows with greater LDT at experiment enrollment would have a greater rate of muscle mobilization across the experimental period was supported. Animals with low LDT at 35 d BEC gained tissue thickness for the first few weeks of the experiment; muscle thickness subsequently began to decrease from one-week BEC until their muscle thickness nadir at 30 DIM. The accretion of muscle suggests the potential for a metabolic target level of muscle mass in cows that is desirable to support fetal growth and lactation. Furthermore, despite showing no statistical difference between 30 and 60 DIM, cows may have mobilized additional LDT between these time points and then reaccreted that tissue by 60 DIM. Cattle with greater muscle thickness, accreted fat between 35 d and 21 d BEC, and significant mobilization of muscle became evident between calving (0 DIM) and 10 DIM. This suggests that LM cows needed to mobilize more muscle than HM cows to support energetic and AA requirements of late gestation. Both groups on average mobilized approximately 26.5% of their 21 d BEC LDT through 30 DIM, which is consistent with the timing but slightly greater in the extent of muscle mobilization previously reported [2,6,14]. The degree of muscle mobilization in both groups was best reflected in 3-MH and the 3-MH:CRE ratio. This is in line with previous research that suggests a strong relationship between muscle mobilization and 3-MH concentrations [6,27]. HM cattle had elevated 3-MH concentrations in late gestation, which suggests an increase in muscle remodeling as there were no phenotypic differences in LDT between groups at 7 d BEC. Furthermore, CRE is a nonenzymatic byproduct of phosphocreatine and creatine breakdown in muscle tissue [28], and it is synthesized at a constant rate in all muscle tissues. However, 3-MH is a marker of muscle mobilization as it is formed during the breakdown of muscle tissue and cannot be resynthesized into skeletal muscle [29]. A previous study documented a moderate positive correlation between LDT and CRE concentrations [21]. As LDT decreased through early lactation, CRE concentrations accordingly did as well, which is consistent with the findings of others [14].

### 4.2. LDT Impact on Adipose Mobilization

The changes in adipose tissue thickness across the study were distinct between HM and LM cattle. At the beginning of the experiments, HM cattle had a greater BCS and BW compared to LM cattle. High-muscle cattle gained BFT during the late dry period to have greater BFT than LM cows at 21 d BEC. Subsequently, HM cows mobilized more BFT over the experiment than LM cows so that there was no difference in BFT, BW, or BCS at 30 and 60 DIM. Although HM cows mobilized more backfat, this was not realized in NEFA concentrations during the four postpartum time points. All cows experienced a significant loss of BCS and bodyweight between 0 and 30 DIM, which is consistent with the timing of adipose mobilization in the literature [2,30,31]. Overall, approximately 15.2% of BFT was lost from 21 d BEC through 30 DIM. Initial BFT in our study was lower than those observed in similar breeds of cattle [31,32,33]. Use of the thoracic vertebrae ultrasound location to evaluate tissue thickness tends to be a smaller tissue reserve than other locations such as those between the *tuber coxae* and *tuber ischiadicum,* or over the transverse process of the lumbar vertebrae [14]. We hypothesized that HM cattle would mobilize more adipose tissue in the form of BFT, which was not found in our current study. Due to a smaller adipose tissue reserve at the thoracic vertebrae, this location may have been less responsive to changes in total body adipose, thus resulting in differences not being detected. The location at the 12th intercostal space was selected due to its ease of accessibility and sensitivity to changes in both *longissimus dorsi* and backfat measurements from parturition to 30 DIM, as previously reported [21]. Moreover, greater changes in BFT measurement may be observed in breeds of cattle that have greater levels of subcutaneous fat [17]. In this study, only subcutaneous fat thickness was measured and so this did not capture the mobilization of other depots such as visceral adipose tissue. This is important to consider, as dairy cows that lost significant retroperitoneal adipose between 1 and 42 DIM exhibited no difference in the average subcutaneous fat deposit between these time points [34].

The amount of LDT or BFT cattle mobilized was related to the respective tissue available at experiment enrollment, which is consistent with previous findings [13,30]. Cows that had the lowest LDT at study enrollment mobilized the least amount of tissue or potentially accreted tissue from 21 d BEC through one month of lactation. Therefore, there may be a lower limit of tissue thickness at which cows do not mobilize.

### 4.3. Enrollment Tissue Thickness Negatively Impacted Production

HM cows produced less milk compared to LM cows through 60 DIM. These cattle also tended to have greater milk fat concentrations although there was no difference in DMI between groups. Previous research found that cattle that were high adipose tissue mobilizers experienced prolonged negative energy balance, reduced intake, had elevated milk fat concentrations, and produced less milk as compared to their herd mates with low or medium levels of mobilization [10,35,36]. While more data collection was needed to fully capture energy balance in this study, significant mobilization of fat was realized in milkfat; however, greater mobilization of LDT was not realized in milk protein, which we initially hypothesized. Previous research on the impact of increased AA supply in early lactation through abomasal infusions of casein or AA profiles of casein found that higher AA supplies increased milk protein and milk lactose output [37,38]. This suggests that cows divert AA towards milk protein synthesis and gluconeogenesis to form lactose. Thus, the higher mobilization of muscle in HM cows may not have resulted in a greater supply of AA to the mammary gland.

In late gestation, instead of storing AA in skeletal muscle, HM cows may have spared them for the developing neonate. Amino acids and glucose flow to the fetus through the process of facilitated diffusion, which drives skeletal muscle synthesis and therefore calf size [39]. The greatest gains in fetal size and AA requirements are observed in the third trimester [1]. Previous work in beef cattle has shown that dams supplemented with protein in the late gestational period gave birth to heavier calves [40]. Additionally, dairy cows that were supplemented with methionine in the late gestational period produced larger male calves, while female calves performed better than a control through weaning [41]. However, these supplemented dams also experienced greater dry matter intake, which may have influenced the amount of nutrients available to the developing fetus.

Our data demonstrate that HM cows gave birth to heavier calves when accounting for the effect of calf sex. Although not directly measured, heavier calves combined with lower milk yields and higher fat content suggest that animals with greater muscle thickness in late gestation may experience a greater degree of insulin resistance in the transition period. Previous studies support this postulate, as cows that mobilized more muscle and fat tissue in early lactation were hypoinsulinemic relative to cows that mobilized less tissue and developed greater insulin resistance to facilitate greater rates of tissue mobilization [10,14,42].

Future work will need to address relationships between LDT and BFT changes over the dry and early lactation period. Previous work has documented a large amount of variability in the type and amount of tissue mobilized over the transition period between cows [43]. While diet and management strategies have been documented to alter tissue reserves through the periparturient period [26,44,45], future work is needed to determine the optimal level of tissue reserves at dry off, or how to obtain the optimal tissue reserves through late gestation and their implications for health and lactation performance.

## 5. Conclusions

The profiles of muscle and fat accretion and mobilization were distinct between multiparous cattle with high versus low muscle thickness at five weeks before expected calving. Cows with greater muscle thickness at five weeks BEC mobilized more muscle and adipose through 30 DIM. Distinct tissue mobilization profiles suggest that metabolic adaptations to the energetic demands of the periparturient period were different and were affected by the amount of initial muscle thickness. High-muscle cows may have partitioned more nutrients towards the developing fetus, thereby leading to larger calves, while a higher mobilization of tissue was associated with decreased milk yield. Future research is needed to understand how muscle and adipose tissue mass and their respective changes in the late gestation and early lactation periods impact dairy cattle milk production, health, and welfare on-farm.

## Figures and Tables

**Table 1:** Nutrient composition for the two rations fed during the two experiments. The prefresh diet was fed from 35 d before expected calving (BEC) to parturition; the lactating diet was fed from 0 to 60 DIM.

Ingredients (g/100 g Diet DM)	First Experiment		Second Experiment	
	Prefresh	Lactating	Prefresh	Lactating
Corn silage	32.7	24.6	31.9	29.8
Mixed mostly legume silage	5.4	19.1	5.3	8.3
Rye hay	16.2	7.8	-	-
Wheat straw	-	-	15.2	6.9
Alfalfa hay	-	-	-	9.3
Whole fuzzy cottonseed	-	6.0	-	-
Dry ground corn	14.4	4.8	14.9	9.6
High moisture corn	-	17.4	-	14.8
Cottonseed hulls	6.6		7.8	-
Soybean meal	6.4	5.5	6.7	5.8
Soyplus ^1^	3.6	4.8	4.3	4.5
Distillers grain with solubles	0.7	-	-	-
LysAAMet ^2^	0.6	0.8	0.7	0.8
Calcium carbonate	1.2	1.1	1.2	1.0
Biochlor ^3^	7.0	-	6.9	-
Vitamin and mineral mix	2.9 ^4^	3.1 ^5^	3.0 ^4^	2.7 ^5^
QLF 63/43 ^6^	2.3	3.7	2.2	4.1
Palmit 80 ^7^	-	1.3	-	2.2
**Nutrient Composition** (% diet DM unless otherwise stated)				
Dry matter (% of diet) ^8^	51.6	53.2	52.9	51.8
Crude protein ^9^	16.9	14.2	15.9	13.8
NDF ^10^	35.5	22.3	37.5	27.2
ADF ^10^	20.5	15.8	24.4	18.4
Ash ^11^	7.8	7.9	5.7	5.1
Starch ^12^	24.6	26.8	24.6	28.7
NFC ^12^	37.2%	42.0%	37.2%	43.3%
Metabolizable energy (Mcal/kg) ^12^	2.40	2.62	2.37	2.58

^1^ Landus Cooperative, Ames, IA, USA; ^2^ 83.0% Blood Meal, 6.8% Smartamine (Adisseo, Alpharetta, GA), 6.8% Biocycle Plus (Agrarian Solutions, Middlebury, IN, USA), and 3.3% AjiPro-L (Ajinomoto Animal Nutrition, Chicago, IL, USA); ^3^ Arm and Hammer Animal Nutrition, Ewing Township, NJ, USA; ^4^ Prefresh vitamin and mineral mix contained 28.55% MegalacR (Church and Dwight Co., Princeton, NJ, USA), 13.96% magnesium oxide, 13.25% Diamond V XP (Diamond V, Cedar Rapids, IA, USA), 9.11% monocalcium phosphate, 8.54% magnesium sulfate, 7.32% salt, 6.68% vitamin E 20,000 IU, 6.07% calcium sulfate, 5.94% mineral premix, 0.43% Rumensin 90 g/lb (40.8 g/kg; Elanco Animal Health, Greenfield, IN, USA), and 0.15% vitamin A; ^5^ Lactating vitamin and mineral mix contained 29.58% sodium bicarbonate, 14.87% salt, 11.89% monocalcium phosphate, 11.71% DCAD Plus (Arm and Hammer), 7.20% magnesium oxide, 6.99% Diamond V XP (Diamond V), 6.54% calcium sulfate, 6.44% fat yellow grease, 3.70% mineral premix, 0.85% vitamin E, and 0.23% Rumensin 90 g/lb (40.8 g/kg; Elanco Animal Health); ^6^ Quality Liquid Feeds, Dodgeville, WI, USA; ^7^ ADM Animal Nutrition, Quincy, IL, USA; ^8^ Determined biweekly via drying at 60 °C for 48 h; ^9^ Determined biweekly based on pure nitrogen (LECO, St. Joseph, MI, USA); ^10^ Samples were analyzed biweekly via the use of neutral and acid detergents (Ankom, Macedon, NY, USA); ^11^ Analyzed biweekly by contents remaining following a 600 °C 24 h oven cycle; ^12^ Obtained from Agriculture Modeling and Training Systems (AMTS; Groton, NY, USA).

**Table 2:** Effect of high-muscle (HM; *n* = 20) and low-muscle (LM; *n* = 21) *longissimus dorsi* thickness (LDT) at 35 d before expected calving (BEC) on body condition score (BCS), bodyweight (BW), and respective changes in tissue thickness from 35 d BEC through 60 d in milk (DIM).

	35 BEC		0 DIM		30 DIM		60 DIM			*p*-Values		
	HM	LM	HM	LM	HM	LM	HM	LM	SEM ^1^	Group ^2^	Day	Group × Day
BCS	3.63 ^a^	3.22 ^c^	3.49 ^b^	3.19 ^c^	2.97 ^d^	2.74 ^e^	2.78 ^e^	2.72 ^e^	0.07	<0.01	<0.01	0.02
BCS Δ per mo.	-	-	−0.05 ^a^	−0.07 ^a^	−0.47 ^b^	−0.45 ^b^	−0.15 ^a^	−0.01 ^a^	0.13	0.13	<0.01	0.06
BW (kg)	780 ^a^	722 ^b^	745 ^b^	699 ^c^	661 ^d^	628 ^d^	655 ^d^	632 ^d^	14.3	0.02	<0.01	0.21
BW Δ (kg)	-	-	−24.2	−28.2	−74.4	−72.5	0.0	−2.5	8.5	0.98	<0.01	0.90
LDT ^3^ Δ per mo. (cm)	-	-	−0.30 ^b,c^	0.51 ^a^	−1.05 ^d^	−0.61 ^c^	−0.18 ^b^	−0.20 ^b^	0.15	<0.01	<0.01	<0.01
Prepartum/Postpartum LDT Δ (cm)	-	-	−0.39 ^b^	0.43 ^a^	-	-	−1.15 ^c^	−0.95 ^c^	0.17	<0.01	<0.01	0.11
Overall LDT Δ (cm)	-	-	-	-	-	-	−1.54	−0.52	0.21	<0.01	-	-
BFT ^4^ Δ per mo. (mm)	-	-	0.65 ^a^	0.03 ^a,b^	−1.18 ^c^	−0.46 ^b,c^	−0.39 ^b,c^	−0.09 ^a,b^	0.30	0.50	<0.01	0.07
Prepartum/Postpartum BFT Δ (mm)	-	-	0.38 ^a^	0.00 ^a,b^	-	-	−1.73 ^c^	−0.68 ^b^	0.34	0.46	<0.01	0.03
Overall BFT Δ (mm)	-	-	-	-	-	-	−0.90	−0.73	0.15	0.73	-	-

^a–e^ Differences in letters indicate a significant difference (*p* < 0.05) between groups by time point interactions; ^1^ SEM determined by largest group × day standard error; ^2^ Cows were assigned to a group based on *longissimus dorsi* thickness (LDT) at 35 d BEC. HM LDT was >4.49 cm and LM LDT was ≤ 4.37 cm at 35 d BEC; ^3^ LDT = *longissimus dorsi* thickness; ^4^ BFT = backfat thickness.

**Table 3:** Effect of high-muscle (HM; *n* = 20) and low-muscle (LM; *n* = 21) *longissimus dorsi* thickness (LDT) at 35 d before expected calving (BEC) on prepartum energy-related metabolites and hormone of insulin, glucose, β-hydroxybutyrate (BHB), non-esterified fatty acids (NEFA), creatinine (CRE), and 3-methyl histidine (3-MH).

Item	Group ^1^			*p*-Values		
	HM	LM	SEM	Group	Day	Group × Day
Insulin (ng/mL) ^2^	0.63	0.56	0.04	0.56	0.24	0.98
Glucose (mg/dL) ^3^	76.8	79.8	0.9	0.03	0.12	0.31
BHB (mmol/L) ^3^	0.49	0.56	0.04	0.26	0.07	0.31
NEFA (mmol/L) ^4^	0.18	0.18	0.03	0.96	<0.01	0.54
CRE (ng/mL) ^2^	3328	3086	94	0.06	0.10	0.29
3-MH (ng/mL) ^2^	453	395	29	0.09	0.14	0.04
3-MH:CRE^2^	0.136	0.121	0.007	0.30	0.15	0.11

^1^ Cows were assigned to a group based on *longissimus dorsi* thickness (LDT) at 35 d BEC. HM LDT was >4.49 cm and LM LDT was ≤4.37 cm at 35 d BEC; ^2^ Metabolite and hormone samples recorded at 35 and 21 d BEC; ^3^ Metabolite samples recorded at 35, 28, 21, 14, and 7 d BEC; ^4^ Metabolite samples recorded at 35, 21, and 7 d BEC.

**Table 4:** Effect of high-muscle (HM; *n* = 20) and low-muscle (LM; *n* = 21) *longissimus dorsi* thickness (LDT) at 35 d before expected calving (BEC) on postpartum energy-related metabolites and hormone of insulin, glucose, β-hydroxybutyrate (BHB), non-esterified fatty acids (NEFA), creatinine (CRE), and 3-methyl histidine (3-MH).

	2 DIM		7 DIM		14 DIM		21 DIM			*p*-Value		
Item	HM	LM	HM	LM	HM	LM	HM	LM	SEM ^1^	Group ^2^	Day	Group × Day
Insulin (ng/mL) ^3^	0.21	0.37	0.18	0.41	0.15	0.31	0.31	0.24	0.10	0.26	0.76	0.27
Glucose (mg/dL) ^3^	68.1	69.8	70.8	64.6	66.3	64.8	69.6	68.8	2.2	0.32	0.17	0.13
BHB (mmol/L) ^3^	0.97	1.04	1.15	1.11	1.08	0.99	0.95	0.87	0.17	0.80	0.48	0.94
NEFA (mmol/L) ^3^	0.64	0.54	0.72	0.62	0.63	0.63	0.75	0.51	0.09	0.15	0.72	0.38
CRE (ng/mL) ^3^	3588	3477	3398	3407	3173	3178	3196	2994	125	0.57	<0.01	0.50
3-MH (ng/mL) ^3^	493 ^a,b,c^	437 ^c,d^	538 ^a^	414 ^d^	581 ^a^	471 ^c^	520 ^a,b^	428 ^c,d^	34	0.02	<0.01	0.17
3-MH:CRE ^3^	0.137 ^c^	0.122 ^c^	0.163 ^b^	0.118 ^c^	0.188 ^a^	0.143 ^b,c^	0.167 ^b^	0.141 ^b,c^	0.010	<0.01	<0.01	0.07

^a–d^ Differences in letters indicate a significant difference (*p* < 0.05) between groups by time point interactions; ^1^ SEM determined by largest group × day standard error; ^2^ Cows were assigned to group based on *longissimus dorsi* thickness (LDT) at 35 d BEC. HM LDT was >4.49 cm and LM LDT was ≤4.37 cm; ^3^ Samples collected at 2, 7, 14, and 21 DIM.

**Table 5:** Relation between high-muscle (HM; *n* = 20) and low-muscle (LM; *n* = 21) *longissimus dorsi* thickness (LDT) at 35 d before expected calving (BEC) on calf birthweight, milk production, and milk components through 60 days in milk (DIM).

Item	HM	LM	SEM	Group *p*-Values ^1^
Calf Birthweight (kg)	45.2	41.8	0.7	<0.01
Milk Yield (kg/d) ^2^	38.8	41.6	0.8	0.02
Fat% ^3^	4.33	4.05	0.12	0.09
Protein% ^3^	2.83	2.88	0.03	0.28
Lactose%^3^	4.75	4.71	0.04	0.42
Fat Yield (g) ^3^	1703	1612	66	0.32
Protein Yield (g) ^3^	1102	1160	34	0.21
Lactose Yield (g) ^3^	1843	1918	57	0.35
Milk Urea Nitrogen (mg/dL) ^3^	7.44	8.08	0.30	0.12

^1^ Cows were assigned to a group based on *longissimus dorsi* thickness (LDT) at 35 d BEC. HM LDT was >4.49 cm and LM LDT was ≤ 4.37 cm at 35 d BEC; ^2^ Calculated from production records from 1 to 60 DIM; ^3^ Calculated from milk samples collected at 7, 14, 21, 30, and 60 DIM.

## Supplementary Materials

The following are available online at https://www.mdpi.com/article/10.3390/ani11082157/s1, Figure S1: Dry matter intake relative to date of calving.

## References

[1] Bauman D.E., Bruce Currie W. (1980). Partitioning of Nutrients During Pregnancy and Lactation: A Review of Mechanisms Involving Homeostasis and Homeorhesis. J. Dairy Sci..

[2] Komaragiri M.V.S., Erdman R.A. (1997). Factors Affecting Body Tissue Mobilization in Early Lactation Dairy Cows. 1. Effect of Dietary Protein on Mobilization of Body Fat and Protein. J. Dairy Sci..

[3] Aschenbach J.R., Kristensen N.B., Donkin S.S., Hammon H.M., Penner G.B. (2010). Gluconeogenesis in dairy cows: The secret of making sweet milk from sour dough. IUBMB Life.

[4] De Koster J., Hostens M., Hermans K., Van den Broeck W., Opsomer G. (2016). Validation of different measures of insulin sensitivity of glucose metabolism in dairy cows using the hyperinsulinemic euglycemic clamp test as the gold standard. Domest. Anim. Endocrinol..

[5] Grummer R.R. (1995). Impact of changes in organic nutrient metabolism on feeding the transition dairy cow. J. Anim. Sci..

[6] Van der Drift S.G.A., Houweling M., Schonewille J.T., Tielens A.G.M., Jorritsma R. (2012). Protein and fat mobilization and associations with serum β-hydroxybutyrate concentrations in dairy cows. J. Dairy Sci..

[7] Drackley J.K. (1999). ADSA Foundation Scholar Award Biology of Dairy Cows During the Transition Period: The Final Frontier?. J. Dairy Sci..

[8] Bell A.W. (1995). Regulation of Organic Nutrient Metabolism During Transition From Late Pregnancy To Early Lactation Efficiency At Pasture View Project. Artic. J. Anim. Sci..

[9] De Koster J.D., Opsomer G. (2013). Insulin Resistance in Dairy Cows. Vet. Clin. N. Am. Food Anim. Pract..

[10] Weber C., Hametner C., Tuchscherer A., Losand B., Kanitz E., Otten W., Singh S.P., Bruckmaier R.M., Becker F., Kanitz W. (2013). Variation In Fat Mobilization During Early Lactation Differently Affects Feed Intake, Body Condition, And Lipid And Glucose Metabolism In High-Yielding Dairy Cows. J. Dairy Sci..

[11] LeBlanc S.J. (2010). Monitoring metabolic health of dairy cattle in the transition period. J. Reprod. Dev..

[12] Grummer R.R. (2008). Nutritional and management strategies for the prevention of fatty liver in dairy cattle. Vet. J..

[13] Pires J.A.A., Delavaud C., Faulconnier Y., Pomiès D., Chilliard Y. (2013). Effects of body condition score at calving on indicators of fat and protein mobilization of periparturient Holstein-Friesian cows. J. Dairy Sci..

[14] Kokkonen T., Taponen J., Anttila T., Syrjälä-Qvist L., Delavaud C., Chilliard Y., Tuori M., Tesfa A.T. (2005). Effect of body fatness and glucogenic supplement on lipid and protein mobilization and plasma leptin in dairy cows. J. Dairy Sci..

[15] Suarez-Trujillo A., Wernert G., Sun H., Steckler T.S., Huff K., Cummings S., Franco J., Klopp R.N., Townsend J.R., Grott M. (2020). Exposure to chronic light–dark phase shifts during the prepartum nonlactating period attenuates circadian rhythms, decreases blood glucose, and increases milk yield in the subsequent lactation. J. Dairy Sci..

[16] McCabe C.J., Suarez-Trujillo A., Teeple K.A., Casey T.M., Boerman J.P. (2021). Chronic prepartum light-dark phase shifts in cattle disrupt circadian clocks, decrease insulin sensitivity and mammary development, and are associated with lower milk yield through 60 days postpartum. J. Dairy Sci..

[17] Greiner S.P., Rouse G.H., Wilson D.E., Cundiff L.V., Wheeler T.L. (2003). The relationship between ultrasound measurements and carcass fat thickness and longissimus muscle area in beef cattle. J. Anim. Sci..

[18] Bruckmaier R.M., Lehmann E., Hugi D., Hammon H.M., Blum J.W. (1998). Ultrasonic measurement of longissimus dorsi muscle and backfat, associated with metabolic and endocrine traits, during fattening of intact and castrated male cattle. Livest. Prod. Sci..

[19] Schwager-Suter R., Stricker C., Erdin D., Künzi N. (2020). Relationship between body condition scores and ultrasound measurements of subcutaneous fat and m. longissimus dorsi in dairy cows differing in size and type. Anim. Sci..

[20] Schäff C., Börner S., Hacke S., Kautzsch U., Sauerwein H., Spachmann S.K., Schweigel-Röntgen M., Hammon H.M., Kuhla B. (2013). Increased muscle fatty acid oxidation in dairy cows with intensive body fat mobilization during early lactation. J. Dairy Sci..

[21] Megahed A.A., Hiew M.W.H., Ragland D., Constable P.D. (2019). Changes in skeletal muscle thickness and echogenicity and plasma creatinine concentration as indicators of protein and intramuscular fat mobilization in periparturient dairy cows. J. Dairy Sci..

[22] NRC (2001). Nutrient Requirments of Dairy Cattle.

[23] Van Soest P.J., Robertson J.B., Lewis B.A. (1991). Methods For Dietary Fiber, Neutral Detergent Fiber, And Nonstarch Polysaccharides In Relation To Animal Nutrition. J. Dairy Sci..

[24] Wildman E.E., Jones I.G.M., Wagner P.E., Boman R.L., Troutt H.F., Lesch T.N. (1982). A Dairy Cow Body Condition Scoring System and Its Relationship to Selected Production Characteristics. J. Dairy Sci..

[25] Iwersen M., Falkenberg U., Voigtsberger R., Foderung D., Heuwieser W. (2009). Evaluation of an electronic cowside test to detect moderate ketosis in dairy cows. J. Dairy Sci..

[26] Zhao H., Wang Y., Yuan B., Liu S., Man S., Xu H., Lu X. (2016). A novel LC-MS/MS Assay For the Simultaneous Determination of Melatonin and Its Two Major Metabolites, 6-Hydroxymelatonin and 6-Sulfatoxymelatonin in Dog Plasma: Application to A Pharmacokinetic Study. J. Pharm. Biomed. Anal..

[27] Doepel L., Lapierre H., Kennelly J.J. (2002). Peripartum Performance and Metabolism of Dairy Cows in Response to Prepartum Energy and Protein Intake. J. Dairy Sci..

[28] Wyss M., Kaddurah-Daouk R. (2000). Creatine and Creatinine Metabolism. Physiol. Rev..

[29] Houweling M., van der Drift S.G.A., Jorritsma R., Tielens A.G.M. (2012). Technical Note: Quantification of Plasma 1- And 3-Methylhistidine in Dairy Cows by High-Performance Liquid Chromatography-Tandem Mass Spectrometry. J. Dairy Sci..

[30] Reynolds C.K., Dürst B., Lupoli B., Humphries D.J., Beever D.E. (2004). Visceral Tissue Mass and Rumen Volume in Dairy Cows during the Transition from Late Gestation to Early Lactation. J. Dairy Sci..

[31] Bünemann K., Von Soosten D., Frahm J., Kersten S., Meyer U., Hummel J., Zeyner A., Dänicke S. (2019). Effects of Body Condition and Concentrate Proportion of the Ration on Mobilization of Fat Depots and Energetic Condition in Dairy Cows During Early Lactation Based on Ultrasonic Measurements. Animals.

[32] Strieder-Barboza C., Zondlak A., Kayitsinga J., Pires A.F.A., Contreras G.A. (2015). Lipid Mobilization Assessment in Transition Dairy Cattle Using Ultrasound Image Biomarkers. Livest. Sci..

[33] Mann S., Nydam D.V., Abuelo A., Leal Yepes F.A., Overton T.R., Wakshlag J.J. (2016). Insulin Signaling, Inflammation, And Lipolysis in Subcutaneous Adipose Tissue of Transition Dairy Cows either Overfed Energy during the Prepartum Period or Fed A Controlled-Energy Diet. J. Dairy Sci..

[34] Akter S.H., Häussler S., Dänicke S., Müller U., von Soosten D., Rehage J., Sauerwein H. (2011). Physiological and Conjugated Linoleic Acid-Induced Changes of Adipocyte Size in Different Fat Depots of Dairy Cows During Early Lactation. J. Dairy Sci..

[35] Roche J.R., Bell A.W., Overton T.R., Loor J.J. (2013). Nutritional Management of The Transition Cow in the 21st Century-A Paradigm Shift in Thinking. Anim. Prod. Sci..

[36] Tamminga S., Luteijn P.A., Meijer R.G.M. (1997). Changes in Composition and Energy Content of Liveweight Loss of Dairy Cows with Time After Parturition. Livest. Prod. Sci..

[37] Larsen M., Galindo C., Ouellet D.R., Maxin G., Kristensen N.B., Lapierre H. (2015). Abomasal Amino Acid Infusion in Postpartum Dairy Cows: Effect on Whole-Body, Splanchnic, and Mammary Amino Acid Metabolism. J. Dairy Sci..

[38] Larsen M., Lapierre H., Kristensen N.B. (2014). Abomasal Protein Infusion In Postpartum Transition Dairy Cows: Effect On Performance And Mammary Metabolism. J. Dairy Sci..

[39] Bell A.W., Slepetis R., Ehrhardt R.A. (1995). Growth and Accretion of Energy and Protein in the Gravid Uterus During Late Pregnancy in Holstein Cows. J. Dairy Sci..

[40] Stalker L.A., Ciminski L.A., Adams D.C., Klopfenstein T.J., Clark R.T. (2007). Effects Of Weaning Date And Prepartum Protein Supplementation On Cow Performance And Calf Growth. Rangel. Ecol. Manag..

[41] Batistel F., Alharthi A.S., Yambao R.R.C., Elolimy A.A., Pan Y.X., Parys C., Loor J.J. (2019). Methionine Supply during Late-Gestation Triggers Offspring Sex-Specific Divergent Changes in Metabolic and Epigenetic Signatures in Bovine Placenta. J. Nutr..

[42] Zachut M., Honig H., Striem S., Zick Y., Boura-Halfon S., Moallem U. (2013). Periparturient Dairy Cows Do Not Exhibit Hepatic Insulin Resistance, Yet Adipose-Specific Insulin Resistance Occurs In Cows Prone To High Weight Loss. J. Dairy Sci..

[43] Komaragiri M.V.S., Casper D.P., Erdman R.A. (1998). Factors affecting body tissue mobilization in early lactation dairy cows. 2. Effect of dietary fat on mobilization of body fat and protein. J. Dairy Sci..

[44] Jaurena G., Moorby J.M. (2017). Lactation and body composition responses to fat and protein supplies during the dry period in under-conditioned dairy cows. J. Dairy Sci..

[45] Douglas G.N., Overton T.R., Bateman H.G., Dann H.M., Drackley J.K. (2006). Prepartal plane of nutrition, regardless of dietary energy source, affects periparturient metabolism and dry matter intake in Holstein cows. J. Dairy Sci..

