# Supplementary figures and images for: Relative Late Gestational Muscle and Adipose Thickness Reflect the Amount of Mobilization of These Tissues in Periparturient Dairy Cattle
Source: Animals (Basel). 2021 Jul 21;11(8):2157. doi: 10.3390/ani11082157 (PMC8388458; doi:10.3390/ani11082157)

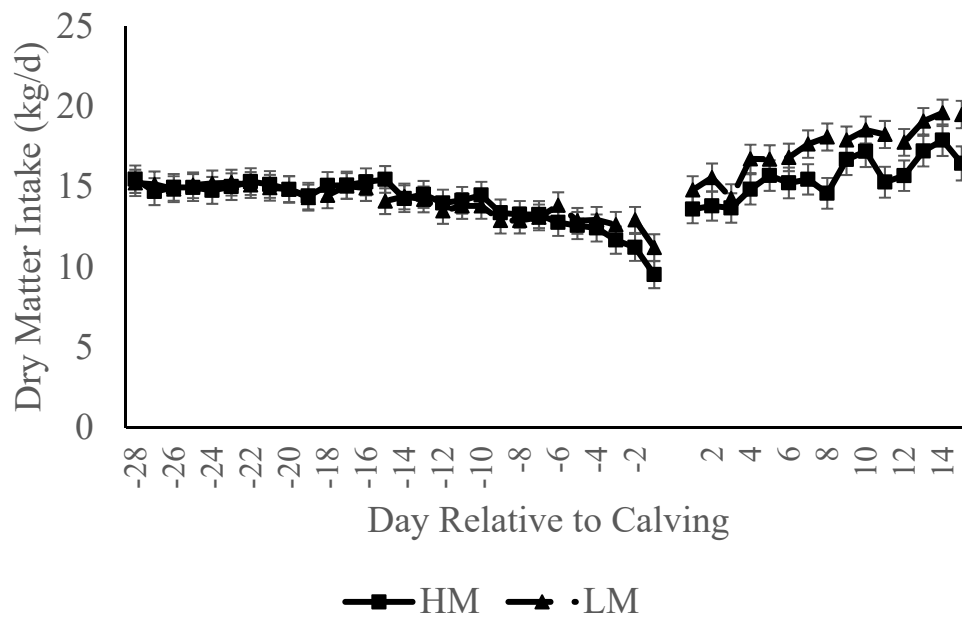

**Figure S1.** Dry matter intake relative to date of calving.

Supplement: Supplementary file 1 [file animals-11-02157-s001.zip › animals-1273034-supplementary.pdf]
